# Minimally Invasive Procedures Dominate American Society of Plastic Surgeons (ASPS)-Reported Procedural Volume in 2024: A Cross-Sectional Descriptive Analysis

**DOI:** 10.7759/cureus.105559

**Published:** 2026-03-20

**Authors:** Saleh Alhotan

**Affiliations:** 1 Department of Surgery, College of Medicine, Qassim University, Buraidah, SAU

**Keywords:** aesthetic surgery, american society of plastic surgeons (asps), minimally invasive procedures, plastic surgery, procedural volume, workforce and workload

## Abstract

Background

National procedural volume summaries are commonly used to describe plastic surgery activity and may inform workforce planning and training exposure. However, the distribution of reported volume across practice categories and the extent of within-category concentration are not well characterized, which limits the interpretation of procedural counts.

Methods

A cross-sectional descriptive secondary analysis of American Society of Plastic Surgeons (ASPS)-reported procedural data for the calendar year 2024 was conducted. Procedures were grouped into three practice categories: cosmetic surgical, minimally invasive, and reconstructive. The total procedural volume was calculated for each category and expressed as a proportion of the overall ASPS activity. Within each category, procedures were ranked by 2024 volume. Concentration was assessed using the Top-5 concentration ratio (CR5), with supplementary analyses using the Top-3 concentration ratio (CR3) and the Herfindahl-Hirschman Index (HHI).

Results

In 2024, the total ASPS procedural volume across cosmetic surgical, minimally invasive, and reconstructive practice categories was 30,877,819 procedures. Minimally invasive procedures accounted for 28,243,407 procedures (91.5% of the total volume), compared with 1,585,878 cosmetic surgical procedures (5.1%) and 1,048,534 reconstructive procedures (3.4%). Minimally invasive volume exceeded cosmetic surgical volume by 26,657,529 procedures and reconstructive volume by 27,194,873 procedures. The five highest-volume procedures accounted for 69.4% of cosmetic surgical volume, 83.6% of minimally invasive volume, and 80.3% of reconstructive volume. Supplementary concentration metrics showed the same pattern, with CR3 values of 52.1%, 67%, and 69.9% and HHI values of 0.1212, 0.1960, and 0.1929, respectively.

Conclusions

The ASPS procedural volume in 2024 was dominated by minimally invasive procedures and was highly concentrated within practice categories. These findings support interpreting procedural totals alongside case mix and within-category concentration rather than relying on procedure counts alone.

## Introduction

The American Society of Plastic Surgeons (ASPS) procedural statistics are widely used as a summary indicator of plastic surgery practice activity in the United States [1]. Hereafter, "ASPS-reported" refers to the aggregated procedural counts reported in annual ASPS statistics. These reports are frequently cited in academic writing and in descriptions of national practice patterns, and they serve as a reference point for how the specialty presents its procedural footprint [1,2]. However, studies rarely examine how reported volume is structured across ASPS practice categories or how much of each category is concentrated within a small set of high-frequency procedures.

Previous analyses drawing on ASPS or closely related aesthetic surgery datasets have mainly focused on long-term trends, economic associations, or changes in procedural popularity over time [2-4]. While this work provides longitudinal context, trend-based summaries may obscure the structural features of reported activity within a single reporting year. In particular, they may underemphasize the relative contribution of minimally invasive, cosmetic surgical, and reconstructive procedures to the total reported volume.

Several data streams suggest strong uptake and expanding visibility of minimally invasive aesthetic procedures. Population-based uptake has been reported in young adult cohorts, and public-interest signals shifted during the COVID-19 period [5,6]. Social media exposure, photograph-editing application use, and appearance dissatisfaction have also been associated with the greater acceptance of cosmetic procedures [7-11]. As a result, minimally invasive procedures may dominate procedure-count summaries even when other categories reflect different clinical demands.

Accordingly, procedural counts require cautious interpretation when readers use volume as a proxy for clinical workload or resource demand. Workforce-planning frameworks in plastic surgery emphasize that procedure counts alone cannot capture differences in case mix, time requirements, or service complexity [12]. At the same time, patient-reported outcome studies suggest that minimally invasive procedures may influence satisfaction with appearance and psychosocial well-being in ways not captured by procedure counts alone [13]. Together, these considerations support a more granular interpretation of what total procedural volume represents.

In this context, this study presents a cross-sectional descriptive analysis of the ASPS procedural statistics for the calendar year 2024. The primary objective was to quantify the contribution of minimally invasive, cosmetic surgical, and reconstructive practice categories to the total reported volume. The secondary objective was to quantify within-category concentration by estimating the proportion of category volume attributable to the five highest-volume procedures. Instead of showing procedure counts in separate tables like the ASPS statistics, this study uses a single method to measure how different categories are made up, how concentrated they are within each category, and how they differ across different practice areas.

## Materials and methods

Study design

This study was a cross-sectional descriptive secondary analysis of ASPS procedural counts for the calendar year 2024 [1]. The analysis was designed to characterize the structure of ASPS procedural activity within a single reporting year rather than evaluate temporal trends.

Data source

Procedure counts were extracted from the publicly available ASPS 2024 Procedural Statistics Release. All procedure-level counts listed in the three ASPS practice-domain tables were reviewed, including cosmetic surgical, minimally invasive, and reconstructive procedures. The dataset consisted of aggregated counts per procedure and did not include patient-level or surgeon-level identifiers.

Unit of analysis and variables

The unit of analysis was the procedure event. Reported counts reflect procedure occurrences rather than unique patients, and a single patient may contribute more than one procedure. Extracted variables included practice category, procedure name as listed in the ASPS report, 2024 procedure count, and source-table inclusion status where applicable.

Data extraction and cleaning

Procedure counts were first compiled in Microsoft Excel (Microsoft Corp., Redmond, WA, USA) and then imported into RStudio (Posit Software, Boston, MA, USA) for analysis. Procedure labels were standardized for brevity and reporting consistency, while counts and practice-domain assignments were retained as reported in the ASPS source tables. Procedures were not combined or reassigned across domains.

For primary domain-level analyses, procedures were retained if they were included in the published ASPS 2024 category totals or listed in the 2024 source tables without an explicit exclusion flag. Rows explicitly marked by ASPS as not included in category totals were excluded from primary calculations.

Derived measures and statistical analysis

The total procedural volume for 2024 was calculated as the sum of cosmetic surgical, minimally invasive, and reconstructive procedure counts. Category contribution was calculated as the proportion of the combined total attributable to each practice domain. Cross-domain comparisons were summarized using absolute differences and fold differences in reported volume.

Within-category concentration was assessed by ranking procedures according to 2024 counts within each domain. The Top-5 concentration ratio (CR5) was defined as the proportion of category volume attributable to the five highest-volume procedures. The Top-3 concentration ratio (CR3) was calculated similarly for the three highest-volume procedures. The Herfindahl-Hirschman Index (HHI) was calculated within each domain as the sum of squared procedure shares, where each procedure share was defined as procedure count divided by total category count. For interpretability, the combined share of the two highest-volume procedures in each domain was also calculated.

Because the analysis used aggregated reported counts, it did not permit inference regarding patient-level exposure, unique patient counts, procedure duration, case complexity, or setting.

Validation and reproducibility

Procedure-level counts were re-summed within each domain and compared against published ASPS totals to validate internal consistency. During revision, all summary tables were regenerated from the cleaned procedure-level dataset after the exclusion of rows explicitly flagged as not included in ASPS category totals.

All calculations and figures were generated using the R statistical software (version 4.5.0; R Foundation for Statistical Computing, Vienna, Austria). Analyses were performed in R using dplyr and ggplot2 with supporting tidyverse dependencies. The code used for data cleaning and analysis is available from the author upon reasonable request.

Presentation of results

Results are presented as counts and percentages without inferential statistical testing, consistent with the descriptive purpose of the study. Because these data represent aggregated reported counts rather than sampled estimates, confidence intervals were not calculated. Bar charts were used to illustrate the distribution of the total ASPS procedural volume across practice categories and within-category procedure concentration.

## Results

Practice category composition of the ASPS procedural volume

**Figure 1:**
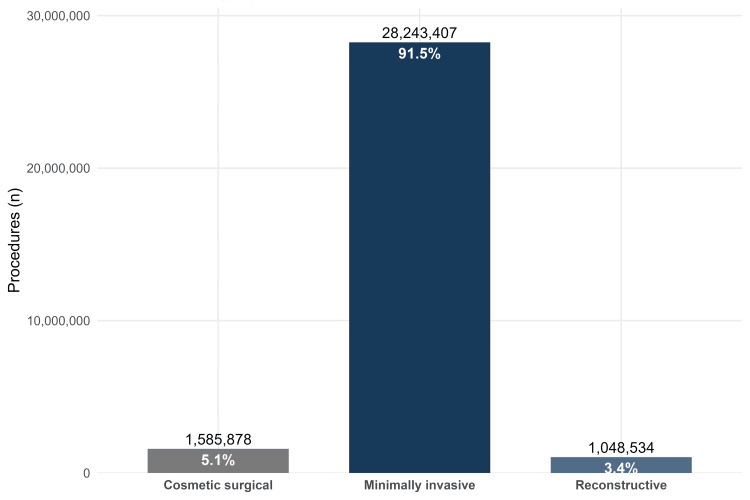
Practice category composition of the ASPS procedural volume in 2024. Distribution of total ASPS procedures across cosmetic surgical, minimally invasive, and reconstructive practice categories in 2024. ASPS: American Society of Plastic Surgeons

In 2024, the total ASPS procedural volume across the three practice categories was 30,877,819 procedures. Minimally invasive procedures accounted for 28,243,407 procedures (91.5% of the total volume). Cosmetic surgical procedures accounted for 1,585,878 procedures (5.1%), and reconstructive procedures accounted for 1,048,534 procedures (3.4%). Minimally invasive volume exceeded cosmetic surgical volume by 26,657,529 procedures and was 17.8-fold higher and exceeded reconstructive volume by 27,194,873 procedures and was 26.9-fold higher (Figure 1).

Procedure concentration within practice categories

**Figure 2:**
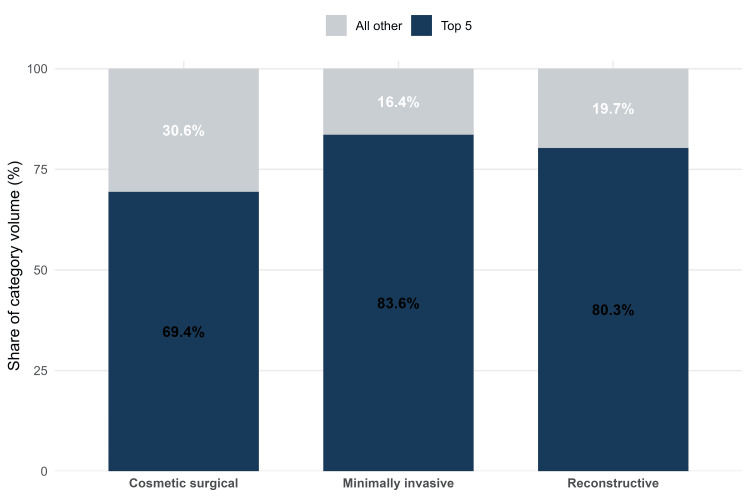
Procedure concentration within ASPS practice categories, 2024. Within each category, the top-5 segments represent the share of category volume attributable to the five highest-volume procedures, and the remaining segment represents all other procedures in that category. The top-5 set differs by category; the category-specific top-5 procedures are listed in the Appendices. ASPS: American Society of Plastic Surgeons

Procedural activity within each practice category was concentrated in a limited number of procedures. In cosmetic surgical practice, the five highest-volume procedures accounted for 1,101,359 procedures, representing 69.4% of category volume, while all remaining cosmetic surgical procedures accounted for 484,519 procedures (30.6%). In minimally invasive practice, the five highest-volume procedures accounted for 23,620,000 procedures, representing 83.6% of category volume, while all remaining minimally invasive procedures accounted for 4,623,407 procedures (16.4%). In reconstructive practice, the five highest-volume procedures accounted for 841,884 procedures, representing 80.3% of category volume, while all remaining reconstructive procedures accounted for 206,650 procedures (19.7%) (Table 1, Figure 2). The leading procedures were liposuction and breast augmentation in cosmetic surgical practice, neuromodulator and hyaluronic acid fillers in minimally invasive practice, and tumor removal and hand surgery in reconstructive practice. The full category-specific top-5 list is provided in the Appendices.

**Table 1:** Within-category procedure concentration in ASPS practice categories, 2024. The top-5 procedures were defined as the five procedures with the highest 2024 counts within each practice category. Percentages represent the share of category volume. ASPS: American Society of Plastic Surgeons

Practice category	Top-5 volume (n)	Top-5 share (%)	All other procedures volume (n)	All other procedures share (%)
Cosmetic surgical	1,101,359	69.4	484,519	30.6
Minimally invasive	23,620,000	83.6	4,623,407	16.4
Reconstructive	841,884	80.3	206,650	19.7

Supplementary concentration metrics

**Figure 3:**
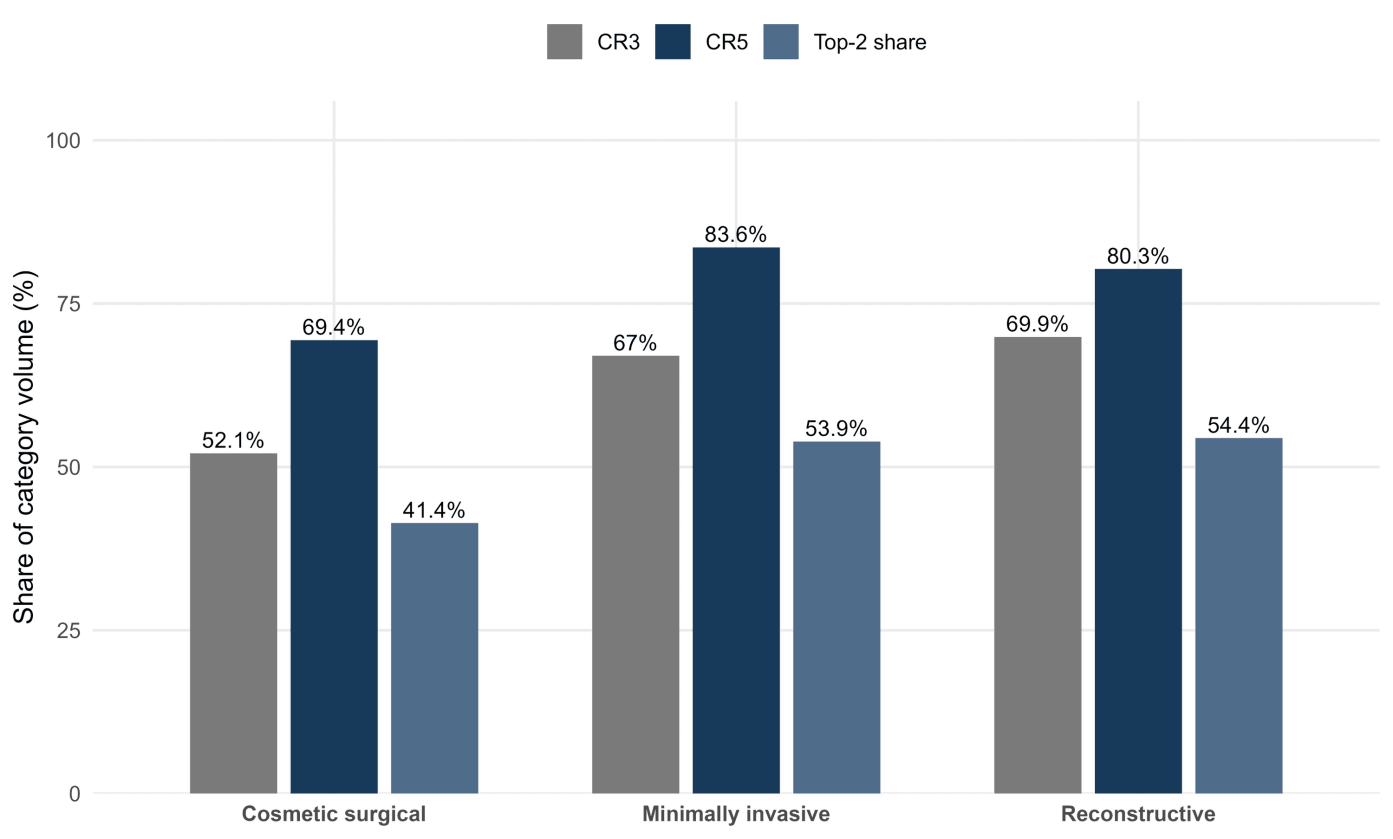
Supplementary concentration metrics by practice category. Bar chart showing the CR3, CR5, and top-2 share for cosmetic surgical, minimally invasive, and reconstructive domains. CR3: Top-3 concentration ratio; CR5: Top-5 concentration ratio

Concentration patterns remained consistent across supplementary metrics (Table 2). The CR3 was 52.1% for cosmetic surgical procedures, 67% for minimally invasive procedures, and 69.9% for reconstructive procedures. The corresponding HHI values were 0.1212, 0.1960, and 0.1929, respectively, indicating greater concentration in minimally invasive and reconstructive domains than in cosmetic surgical practice. The two highest-volume procedures in each domain also accounted for a substantial share of category activity: liposuction and breast augmentation represented 41.4% of cosmetic surgical volume, neuromodulator and hyaluronic acid fillers represented 53.9% of minimally invasive volume, and tumor removal and hand surgery represented 54.4% of reconstructive volume (Figure 3).

**Table 2:** Supplementary concentration metrics by practice category. CR3: Top-3 concentration ratio; CR5: Top-5 concentration ratio; HHI: Herfindahl-Hirschman Index

Practice category	Number of procedures	CR3 (%)	CR5 (%)	HHI	Top-2 share (%)
Cosmetic surgical	25	52.1	69.4	0.1212	41.4
Minimally invasive	11	67	83.6	0.1960	53.9
Reconstructive	15	69.9	80.3	0.1929	54.4

## Discussion

This study examined the structure of ASPS procedural activity in 2024, with emphasis on practice-category composition and within-category concentration. Two principal findings emerged. First, minimally invasive procedures accounted for the large majority of reported procedural volume. Second, procedural activity within each practice category was concentrated in a small number of procedures. These findings are consistent with the ASPS 2024 procedural statistics and with prior reporting that a limited set of procedures accounts for a substantial share of recorded plastic surgery activity [1,2].

In 2024, minimally invasive procedures represented more than 90% of all ASPS procedures, whereas cosmetic surgical and reconstructive procedures together accounted for less than 10% of the total reported volume. This pattern is consistent with the high frequency of treatments such as neuromodulators, fillers, skin resurfacing, and laser-based procedures in the ASPS 2024 statistics [1]. It is also broadly consistent with international aesthetic survey reporting showing that non-surgical procedures contribute heavily to total procedure counts [14]. However, these counts should be interpreted as frequency measures rather than as direct measures of treatment burden, visit complexity, or care setting [12].

A limited number of procedures dominated volume within each practice category. The five highest-volume procedures accounted for 69.4% of cosmetic surgical activity, 83.6% of minimally invasive activity, and 80.3% of reconstructive activity. Supplementary concentration metrics supported the same pattern: CR3 was 52.1%, 67%, and 69.9%, respectively, and HHI values were higher for minimally invasive and reconstructive domains than for cosmetic surgical practice. Together, these findings indicate that reported procedural activity was not evenly distributed across procedure types but instead organized around a limited procedural core within each category. Prior ASPS-based practice-profile work similarly emphasizes that a relatively small set of procedures drives a large share of recorded activity [2].

These findings have important implications for the interpretation of procedural statistics. High procedure counts for minimally invasive care should not be interpreted as equivalent to a greater workload or resource demand than lower-volume surgical or reconstructive care. Procedure frequency does not directly reflect operative time, staffing requirements, case complexity, facility use, or perioperative resource utilization [12]. Reported counts also reflect procedures rather than unique patients. Accordingly, volume-based summaries should be interpreted cautiously when used to infer workforce demands or training exposure [12].

At the same time, procedure counts alone do not capture the full clinical meaning of aesthetic practice. Patient-reported outcome literature suggests that minimally invasive procedures may affect satisfaction with appearance and psychosocial well-being in ways not reflected by aggregate procedural volume [13]. Accordingly, high-frequency procedures may have importance beyond what volume counts alone convey, even though this dataset cannot evaluate outcomes directly [13].

The present analysis describes the structural features of procedural volume but does not test causal mechanisms underlying procedure demand. While prior literature suggests that digital visibility and social media exposure may influence interest in aesthetic interventions [6-8,11], these mechanisms cannot be evaluated using aggregated procedural counts. Any external factors contributing to the observed concentration of minimally invasive activity remain hypothesis-generating and fall outside the scope of this descriptive dataset.

This study has several limitations. First, the analysis used aggregated ASPS procedural data for a single calendar year and therefore cannot assess temporal stability or change over time. Second, the dataset captured procedures rather than unique patients and did not include outcomes, case complexity, procedure duration, reimbursement, or care setting. Third, the analysis depended on ASPS procedure definitions, reporting structure, and domain-specific inclusion rules. In addition, the ASPS minimally invasive procedure table includes data from ASPS members as well as non-member dermatologists and otolaryngologists, which may influence comparisons of category proportions across practice domains. Finally, as a secondary descriptive analysis of published counts, the findings are limited to the structural features of the reported 2024 dataset and should not be generalized automatically to other datasets or specialties.

Despite these limitations, this study provides a structured overview of how ASPS procedural activity was organized in 2024. By separating practice-category composition from within-category concentration and confirming the same pattern across CR3, CR5, and HHI, the analysis adds a structured interpretive framework for understanding aggregate procedural statistics and supports more careful use of procedure counts when describing plastic surgery activity.

## Conclusions

The majority of the ASPS procedural volume in 2024 came from minimally invasive procedures, and a small number of procedures accounted for most of the volume within each practice category. The ASPS procedural statistics provide a useful overview of reported national procedural activity. However, total counts should be interpreted alongside case mix and within-category concentration, because procedure counts alone do not capture workload, complexity, or resource use.

## Appendices

**Table 3:** Top-5 procedures by volume within each ASPS practice category in 2024. The top-5 procedures were defined as the five procedures with the highest reported volume within each practice category for the calendar year 2024, based on the ASPS annual procedural statistics. HA: hyaluronic acid; ASPS: American Society of Plastic Surgeons

Practice category	Procedure	Procedures in 2024	Rank within category
Cosmetic surgical	Liposuction	349,728	1
Cosmetic surgical	Breast augmentation	306,196	2
Cosmetic surgical	Abdominoplasty	171,064	3
Cosmetic surgical	Breast lift	153,616	4
Cosmetic surgical	Eyelid surgery	120,755	5
Minimally invasive	Neuromodulator	9,883,711	1
Minimally invasive	HA fillers	5,331,426	2
Minimally invasive	Skin resurfacing	3,703,305	3
Minimally invasive	Skin treatment using lasers	3,112,056	4
Minimally invasive	HA fillers for lip augmentation	1,589,502	5
Reconstructive	Tumor removal	361,798	1
Reconstructive	Hand surgery	208,480	2
Reconstructive	Breast reconstruction	162,579	3
Reconstructive	Maxillofacial	54,747	4
Reconstructive	Scar revision	54,280	5
